# Evaluation of *Ulva lactuca* hydrolysates as a feedstock for clostridial fermentations to produce 1,2-propanediol

**DOI:** 10.3389/fmicb.2026.1730238

**Published:** 2026-03-27

**Authors:** Eva Maria Ingvadottir, Sean M. Scully, Johann Orlygsson

**Affiliations:** Faculty of Natural Resource Sciences, University of Akureyri, Akureyri, Iceland

**Keywords:** 1,2-propanediol, bioprocessing, chiral molecules, macroalgae, methylpentose, moderate thermophiles, seaweed, third generation biomass

## Abstract

Macroalgae are an abundant and underutilized renewable feedstock that can be exploited for the production of various low- and high-value biomolecules. The study herein describes a mild acid and base process to hydrolyze the green macroalgae *Ulva lactuca* into simpler, fermentable carbohydrates, with an emphasis on rhamnose. Hydrolysis experiments involving up to 5% v/v sulfuric acid and 5% w/v sodium hydroxide at temperatures between 25 and 100 °C demonstrated that the highest recovery of fermentable carbohydrates was generally obtained using 2.5% v/v sulfuric acid at 75 °C. Two *Clostridium* species (*Clostridium* strain AK1 isolated from SW Iceland, and *Clostridium beijerinckii* strain DSM 791) were used to ferment L-rhamnose to 1,2-propanediol, both as a single substrate and as part of macroalgal hydrolysates. Additionally, the impact of culture conditions (pH and initial substrate concentration) on rhamnose fermentation was investigated in batch culture for both strains. Generally, pH did not influence the production of 1,2-propanediol and both strains partially degraded rhamnose at very low (10 mM) initial substrate concentrations. A kinetic investigation of rhamnose utilization using strain AK1 showed that the pentose is degraded much slower as compared with glucose with 1,2-propanediol production lagging and reaching a maximum concentration of 7.7 mM. When *Clostridium* strain AK1 was cultivated on *U. lactuca* hydrolysates and non-pretreated *U. lactuca*, the maximum yields were 7.9 mM 1,2-propanediol. This is the first report of the production of 1,2-propanediol from macroalgal biomass using a moderately thermophilic Clostridia.

## 1 Introduction

The quest for blue bio-based economies has brought attention to the biotechnological potential of macroalgae, more commonly referred to as seaweed. While humans have utilized edible seaweeds since antiquity (Suryanarayana Murty and Banerjee, 2011), their use as sources of natural products such as novel pharmaceuticals with desirable bioactivities dates back decades. However, their utility as feedstock for biofuel production as an alternative to first- and second-generation feedstocks is relatively recent. As arable land and inexpensive fertilizers become scarcer, the importance of macroalgae will only continue to increase.

In the context of bioprocessing, it is noteworthy that many macroalgae can contain up to 75% dry weight carbohydrates (Jung et al., 2013; Wu et al., 2014) although the composition of this fraction largely depends on growth conditions (Misurcová, 2012). Due to their high polysaccharide content, fast growth ratio, and the fact that macroalgae do not require arable land for growth, they present a valuable and underutilized renewable feedstock for third generation biofuels. The bioprocessing of macroalgae, however, is more challenging than lignocellulosic biomass given the more complex nature of the carbohydrate fraction, consisting of both a greater number of sulfated polysaccharides and monosaccharide building blocks. Examples include fucose-rich fucoidan, alginate, galactopyranose-based carrageenans, rhamnose-rich ulvan, and glucans such as laminarin, cellulose, and starch (Singh et al., 2024).

*Ulva lactuca*, colloquially known as “sea lettuce,” is an edible green seaweed which tends to be a nuisance organism as its growth and decay can lead to environmental problems and economic loss (Dominguez and Loret, 2019). As such, it presents an inexpensive and abundant source of biomass for bioprocessing. *U. lactuca*’s global distribution, including the shorelines of Iceland, makes it a potentially abundant and easily harvested source of biomass for the biotechnological production of biofuels and other useful bio-based molecules. *Ulva* species, such as *U. lactuca* and *U. intestinalis*, contain a large fraction of fermentable polysaccharides such as starch and ulvan, the latter of which is a heteropolysaccharide composed of sulfated L-rhamnose residues (Tsubaki et al., 2016) which can be fermented to (*S*)-1,2-propanediol (1,2-PD). This three carbon, chiral alcohol has found applications across industries due to its low toxicity and solvent properties. Examples include drug and cosmetic formulations, foods, and deicing fluids (Tao et al., 2021). Additionally, its chiral nature has made it a useful pharmaceutical building block; (*R*)-1,2-propanediol is one of the starting materials in the synthesis of the antibiotic Levofloxacin (Laneman, 2006).

There are two main biological pathways leading to the production of 1,2-PD. The first involves the degradation of deoxysugars, also called methylpentoses, such as L-fucose and L-rhamnose, to yield the *S-*enantiomer via reduction of L-lactaldehyde (Boronat and Aguilar, 1981). This deoxyhexose pathway has been reported in a number of facultative, mesophilic anaerobes including *Escherichia coli*, *Salmonella typhimurium*, *Klebsiella pneumoniae* (Badía et al., 1985), *Paenibacillus macerans* (Weimer, 1984), as well as obligate anaerobes such as *Bacteroides ruminicola* (Turner and Anthony, 1979), *Clostridium beijerinckii* (Bikker et al., 2016; Diallo et al., 2019), *Clostridium phytofermentans* (Petit et al., 2013), *Clostridium* strain AK1 (Ingvadottir et al., 2018), and *Caldicellulosiruptor* spp. (Ingvadottir et al., 2017). *Clostridium* strain AK1 is particularly interesting as it is a highly stenothermal, ethanologenic, moderate thermophile isolated from a remote geothermal field in the Southwest of Iceland (Orlygsson, 2012). As moderate thermophiles are underrepresented in the literature, this strain offers a different approach to producing 1,2-PD from biomass-derived rhamnose.

The second pathway leading to 1,2-PD is the methylglyoxal bypass pathway which proceeds through the glycolytic intermediate dihydroxyacetone phosphate (DHAP) via formation of methylglyoxal and its subsequent reduction to D-lactaldehyde or acetol and finally *R-*enantiomer of 1,2-PD. Reports of wild type (*R*)-1,2-PD producers from D-sugars such as D-glucose in the literature are limited to *Clostridium sphenoides* under phosphate limiting conditions (Tran-Din and Gottschalk, 1985), *Thermoanaerobacterium thermosaccharolyticum* strain HG-8 (Altaras et al., 2001; Cameron and Cooney, 1986; Sanchez-Riera et al., 1987), and *Clostridium thermobutyricum* (Enebo, 1954).

Several species within Class *Clostridia* have demonstrated a propensity for the utilization of algal components. This includes the fermentation of pretreated brown, red, or green algae by *Thermoanaerobacter* spp. (Chades et al., 2018), *Clostridium beijerinckii* (Diallo et al., 2019; Hou et al., 2017), *Clostridium tyrobutyricum* (Oh et al., 2019), and *Clostridium acetobutylicum* (Schultze-Jena et al., 2022), with end products typically being dominated by butyric acid, acetone, butanol, ethanol, and hydrogen. However, the utilization of methylpentoses from green and brown macroalge to produce enantiomerically pure 1,2-PD seems to remaina ubiquitous and mostly unexplored facet of clostridial metabolism.

The aim of this work was to investigate the ability of the moderately thermophilic *Clostridium* strain AK1 to ferment *U. lactuca* hydrolysates to produce 1,2-PD. The pretreatment of *U. lactuca* was evaluated with emphasis on L-rhamnose extraction prior to fermentation using *Clostridium beijerinckii* (DSM 791) for comparison. Furthermore, the effect of pH and initial L-rhamnose concentration on end product formation of both organisms was investigated.

## 2 Materials and methods

### 2.1 Strains and cultivation conditions

Two clostridia species, *Clostridium* strain AK1 (DSM 18778) and *Clostridium beijerinckii* (DSM 791) were cultivated in Basal Mineral (BM) medium prepared as previously described (Chades et al., 2018) using the Hungate technique (Hungate, 1969; Miller and Wolin, 1974). BM medium was autoclaved for 1 h at 121 °C; other components were syringe filtered through 0.45 μm filters. Fermentations were conducted at pH 7.0 (adjusted with 1 M HCl or NaOH) and a liquid–gas phase ratio of 1:1; *Clostridium beijerinckii* was cultivated at 35 °C and *Clostridium* strain AK1 at 50 °C. Inoculation volumes were 2% (v/v) taken from cultures in the exponential growth phase (cultivated on 20 mM glucose) prepared from frozen (−20 °C) cultures stored in rigorously degassed BM medium containing 30% (v/v) glycerol. Fermentations were routinely performed in either serum bottles or Hungate tubes (ChemGlass, UK) fitted with butyl rubber septa. All cultivations were performed in triplicate and cultures grown for 7 days after which they were analyzed for end product formation.

### 2.2 Collection and preparation of *Ulva lactuca*

*U. lactuca* was harvested from the Húsavík coastline (north central Iceland, 66°3′38.64′′N, 17°21′18.92′′W) at low tide in June of 2016. Biomass was transported back to the laboratory, briefly rinsed with cold tap water, and dried at 45–50 °C for 48 h. Dry *U. lactuca* was then milled using a Waring blender and stored at ambient temperature in an airtight container prior to use.

### 2.3 Evaluation of pretreatment on *Ulva lactuca* hydrolysis

Dry *U. lactuca* biomass was treated at 25, 50, 75, and 100 °C using either sulfuric acid (v/v) or sodium hydroxide (w/v) solutions including 0.0, 0.1, 0.5, 1.0, 2.5, and 5.0%. dH_2_O served as a control. One milliliter of sulfuric acid or sodium hydroxide solution was added to 100 mg of biomass in a 13 × 100 mm culture tube and incubated for 1 h in a temperature-controlled water bath. Five hundred microliter of solution were then removed and centrifuged at 13,000 rpm for 3 min prior to analysis.

### 2.4 Kinetics of *Ulva lactuca* hydrolysis

Based upon the results obtained from pretreatment evaluation (see Section 2.3), hydrolysis kinetics were investigated at 75 °C using either dH_2_O or sulfuric acid (0.5, 1, or 2.5% v/v) as the hydrolysis medium. Five milliliter of solution were added to 500 mg of dried biomass in an 18×150 mm culture tube and incubated for 350 min in a temperature-controlled water bath. One hundred and fifty microliter samples were periodically (9 times in total) withdrawn and frozen prior to analysis.

### 2.5 Preparative-scale hydrolysis of *Ulva lactuca*

Based upon the results obtained from hydrolysis kinetics (see Section 2.4), *U. lactuca* biomass was hydrolysed on a preparative scale using 25 g of dry macroalgae and 250 mL of extraction solution (dH_2_O or 1% v/v H_2_SO_4_) and heated at 75 °C for 3 h. Solid material was removed by centrifugation (4,700 rpm, 20 min) and the supernatant collected, adjusted to pH 7.0, and a V_f_ of 250 mL with dH_2_O.

### 2.6 Fermentation of model substrates

The ability of the two clostridia strains to utilize model substrates was evaluated in batch culture in 25 mL serum bottles with initial substrate concentrations of 20 mM for monomeric compounds and 2% (w/v) for polymeric substrates; this included glucose, rhamnose, an even mixture of glucose and rhamnose, starch, and alginate. It should be noted that polymeric substrates were added directly to the BM prior to autoclaving.

### 2.7 Influence of culture conditions on L-rhamnose fermentations

The influence of initial rhamnose concentration and pH on growth and 1,2-PD formation of both clostridia strains was evaluated in batch culture. Initial rhamnose concentration was evaluated between 10 and 120 mM; the influence of pH was evaluated at a concentration of 20 mM for rhamnose from pH 4.5 to 8.5 in 0.5-unit increments. The pH was adjusted using 1 or 6 M HCl and NaOH. Fermentations were performed in 16 × 150 mm Hungate tubes which were incubated for 14 days after which end products were analyzed.

### 2.8 Fermentation of *Ulva lactuca* hydrolysates

Bm medium containing 5–70% (v/v) of the macroalgal hydrolysate (see Section 2.5) or 10% (w/v) *U. lactuca* meal was prepared in 8.7 mL serum bottles and sterilized by Tyndallization (95 °C for 1 h, 25 °C for 24 h, 95 °C for 1 h). Cultures were incubated for 14 days after which end products were analyzed.

### 2.9 Fermentation kinetics of model substrates by *Clostridium* strain AK1

Strain AK1 was cultivated on glucose (20 mM), rhamnose (20 mM) and a mixture of glucose and rhamnose (both 20 mM) for a period of two weeks (336 h). Substrate and degradation rates were calculated based on substrate consumption and product formation by analyzing metabolite concentrations collected at different time intervals based on moles degraded and formed.

### 2.10 Analytical methods

Hydrogen was quantified via GC-TCD and carboxylic acids and alcohols by GC-FID as previously described by Orlygsson and Baldursson (2007). Optical density was obtained at 600 nm using a Shimadzu UV-1800 UV–Visible spectrophotometer with a pathlength of 1 cm against a dH_2_O blank. Cell-free solutions were prepared by centrifugation at 13,000 rpm (3 min) prior to analysis.

Total reducing sugars were analyzed colorimetrically using the 3,5-dinitrosalicylic acid (3,5-DNS) method described by Miller (1959) with minor modifications. Briefly, 100 μL of sample and 100 μL of 3,5-DNS reagent (1% w/v 3,5-DNS, 4% w/v Na_2_SO_3_, and 4% w/v NaOH in dH_2_O) were added to a microtiter plate and incubated at 90 °C for 20 min. After cooling to ambient temperature, 33 μL of tartaric acid (40% w/v) were added and the microplate shaken at 100 rpm for 30 s prior to reading on a Bioscreen C (Growth Curves Ltd., Finland) at 580 nm. Glucose solutions ranging from 1 to 30 mM were used as a standard.

Methylpentoses were analyzed colorimetrically according to the method of Dische and Shettles (1948) with minor modifications for a microtiter plate format. Fifty microliter of sample and 225 μL of sulfuric acid (15.4 M) were added to a microtiter plate and incubated at 90 °C for 20 min. The plate was cooled to ambient temperature and 10 μL of cysteine-HCl solution (3% w/v) added. The plate was then shaken at 100 rpm for 30 s and incubated at ambient temperature for 1 h at which time the plate was read on a Bioscreen C (Growth Curves Ltd., Finland) at 580 nm. L-rhamnose solutions ranging from 0.1 to 3 mM were used as a standard.

Protein was quantified using the Bradford method (Bradford, 1976). Briefly, 300 μL of Bradford reagent (100 mg of Coomassie Brilliant Blue G-250 suspended in 1:2:17 ethanol/H_3_PO_4_/dH_2_O) was added to a microtiter plate containing 50 μL of sample. The plate was briefly shaken (100 rpm, 30 s) and immediately read at 600 nm. Bovine serum albumin was used to prepare standards ranging from 0.1 to 1.4 mg/mL.

Starch was quantified using the methodology of Gur et al. (1969). Briefly, 50 μL of sample and 200 μL of a I_2_-KI reagent (aqueous solution consisting of 2.5 mM I_2_ and 5 mM KI) were added to a microtiter plate and read on a Bioscreen C (Growth Curves Ltd., Finland) at 600 nm. Starch solutions ranging from 0–900 mg/L were used as standards.

1,2-PD was analyzed colorimetrically according to the method described by Jones and Riddick (1957) with modifications. Briefly, 50 μL of sample was added to a microtiter plate followed by 250 μL of concentrated sulfuric acid and incubation at 70 °C for 20 min. After cooling to room temperature, 10 μL of a ninhydrin solution (3 g ninhydrin and 5 g NaHSO_4_ dissolved in 100 mL dH_2_O) were added and the plate incubated for 60 min at room temperature before reading on a Bioscreen C (Growth Curves Ltd., Finland) at 600 nm. 1,2-PD standards were prepared at a concentration range of 0.1–2 mM.

Total phenolics were analyzed colorimetrically according to the method described by Singleton and Rossi (1965) with modifications. Briefly, 50 μL of sample, 200 μL of dH_2_O and 25 μL of Folin–Ciocalteu Reagent (Sigma F9252) were added to a microtiter plate followed by 75 μL of a 20% (w/v) Na_2_CO_3_ solution and 100 μL of dH_2_O. The plate was allowed to develop over 2 h and read on a Bioscreen C (Growth Curves Ltd., Finland) at 600 nm. Gallic acid standards were prepared at a concentration range of 5–100 mg/L. Results are presented as gallic acid equivalent (GAE).

### 2.11 Statistical analysis

Two-tailed paired Student’s *t*-tests (95% confidence interval), Pearson’s correlation coefficient (*r*), one-way ANOVA, and Tukey’s honest significance tests (95% confidence interval) were calculated using Microsoft Excel.

## 3 Results

### 3.1 Evaluation of pretreatment on *Ulva lactuca* hydrolysis

**Figure 1:**
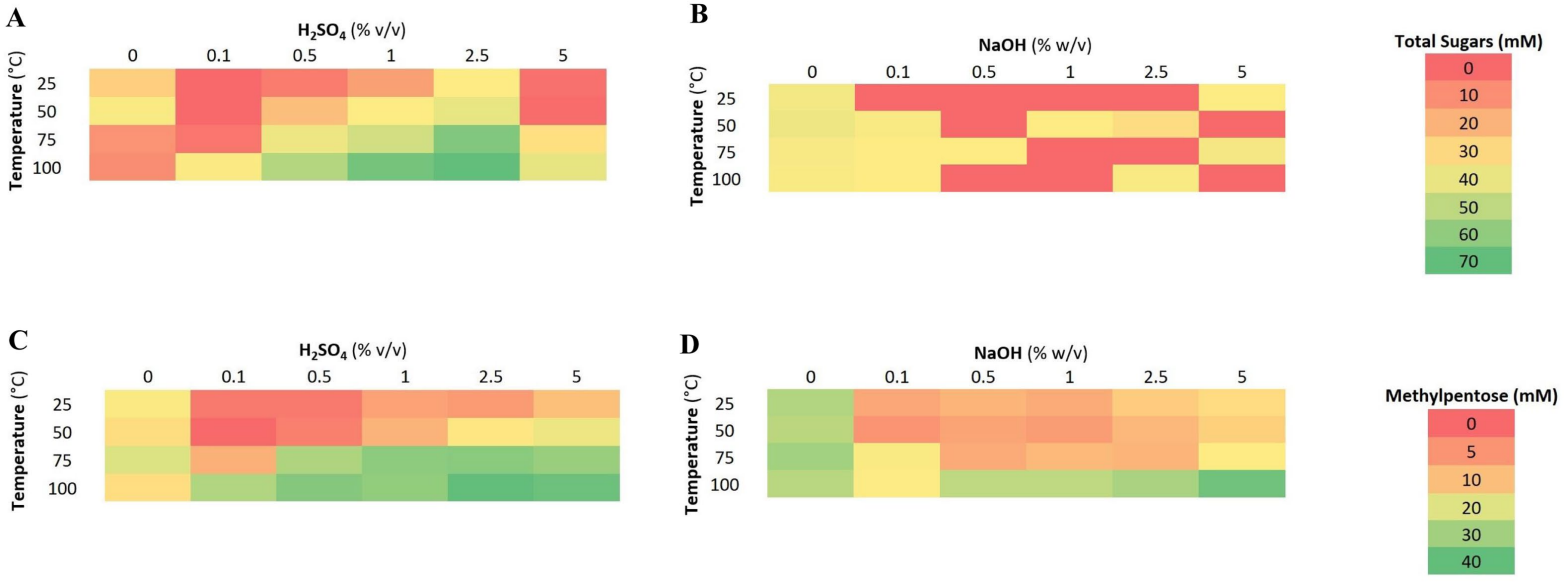
Release of total sugars, methylpentoses, and phenolic conditions during acidic **(A,C)** and alkaline (conditions) **(B,D)** in mM. As phenolic compounds are undesirable, increased concentrations are highlighted red as opposed to increasing concentrations of total sugars and methylpentoses which are highlighted green.

Under acidic conditions, the amount of liberated total sugars ranged from 2.2 mM (0.1% v/v H_2_SO_4_ at 25 °C) glucose equivalence to 61.8 mM (2.5% v/v H_2_SO_4_ at 100 °C). Generally, an increase in both temperature and acid concentration led to an increase in liberated reducing sugars although a drop in sugar release was observed at the highest concentration of sulfuric acid (5% v/v). A similar trend was observed with the amount of liberated methylpentoses (which ranged from 8.4 to 39.3 mM using 2.5% sulfuric acid at 100 °C) and total phenolics (48.9–441.5 mg/L) as presented in Figure 1.

Under alkaline conditions, the amount of liberated reducing sugars reached a maximum of 3.2 mM (0.1% v/v NaOH at 50 °C). Highest concentrations of methylpentoses (37.0 mM) were observed when using 5% NaOH at 100 °C accompanied by a high release of total phenolics (656.3 mg/L) as summarized in Figure 1.

### 3.2 Kinetics of *Ulva lactuca* hydrolysis

To better evaluate the time needed to achieve maximum rhamnose liberation, *U. lactuca* meal was extracted under four different acid concentrations (0, 0.5, 1, and 2.5% v/v) at 75 °C and periodically sampled over a period of 6 h. 75 °C were chosen as most rhamnose was generally observed to be released at this temperature.

**Figure 2:**
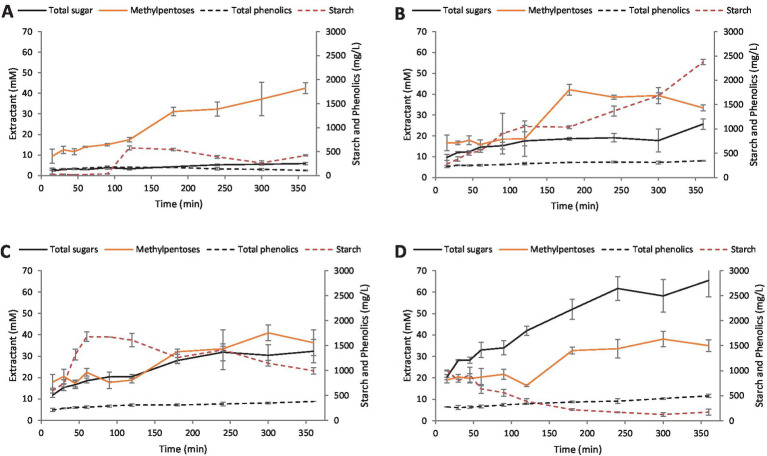
Extraction of sugars from *Ulva lactuca* at 75 °C. **(A)** dH_2_O, **(B)** 0.5% v/v sulfuric acid, **(C)** 1% v/v sulfuric acid, **(D)** 2.5% v/v sulfuric acid. Values represent the average of triplicates with standard deviation presented as error bars.

A control extraction of macroalgal biomass using dH_2_O at 75 °C showed a total sugar concentration of 2.2 mM after 15 min which gradually increased to 5.9 mM after 6 h (Figure 2A). Similarly, the methylpentose assay suggested that rhamnose content slowly increased, reaching a value of 42.5 mM after 6 h. Also, a general increase in the amount of solubilized starch was observed, ranging from 23.5 mg/L after 15 min up to 578.5 mg/L after 180 min before decreasing to 418.5 mg/L at 6 h. The total phenolic content of the hydrolysate stayed below 200 mg/L over the 6 h extraction period.

Treating *U. lactuca* with a 0.5% v/v sulfuric acid solution yielded higher concentrations of total sugars on average, although the amount of liberated methylpentoses did not increase (Figure 2B). Concentration of total sugars generally increased to 18.6 mM after 3 h and reached 25.7 mM after 6 h. The concentration of methylpentoses reached a maximum of 42.1 mM at 3 h but decreased to 33.4 mM after 6 h. The amount of starch present after 2 h was 1049.8 mg/L and then gradually increased to 2374.8 mg/L after 6 h while the amount of phenolics reached a maximum of 345.9 mg/L at the time, more than threefold as compared with treatment without acid.

The use of 1% v/v sulfuric acid generally resulted in a more rapid liberation of starch and subsequent conversion to reducing carbohydrates as compared with the water control and lower acid concentrations (Figure 2C). Under these conditions, starch reached a maximum of 1677.3 mg/L after 1 h and declined over the duration of the experiment while the concentration of total sugars increased from 11.8 mM to that of 31.9 mM after 4 h before increasing to a maximum of 32.3 mM at 6 h. Similarly, the methylpentose concentration reached 40.9 mM at 5 h and then decreased slightly to 36.3 mM at 6 h. Under these conditions, the total phenolic concentration roughly doubled from 207.0 mg/L to 378.0 mg/L after 6 h.

Finally, the use of 2.5% v/v sulfuric acid for the hydrolysis of *U. lactuca* resulted in the highest concentration of total sugars liberated on average (Figure 2D). The initial concentration of solubilized starch was 1005.6 mg/L (at 15 min) and rapidly declined to 388.1 mg/L after 2 h while the amount of total sugars increased from 20.4 mM to 42.0 mM. The total sugar concentration reached 61.7 mM after 4 h and then increased slightly to 65.4 mM (after 6 h). The concentration of methylpentoses reached a concentration of 38.1 mM after 5 h but slightly decreased to 35.0 mM after 6 h. The total phenolics concentration reached 497.6 mg/L after 6 h.

### 3.3 Fermentation of model substrates

**Figure 3:**
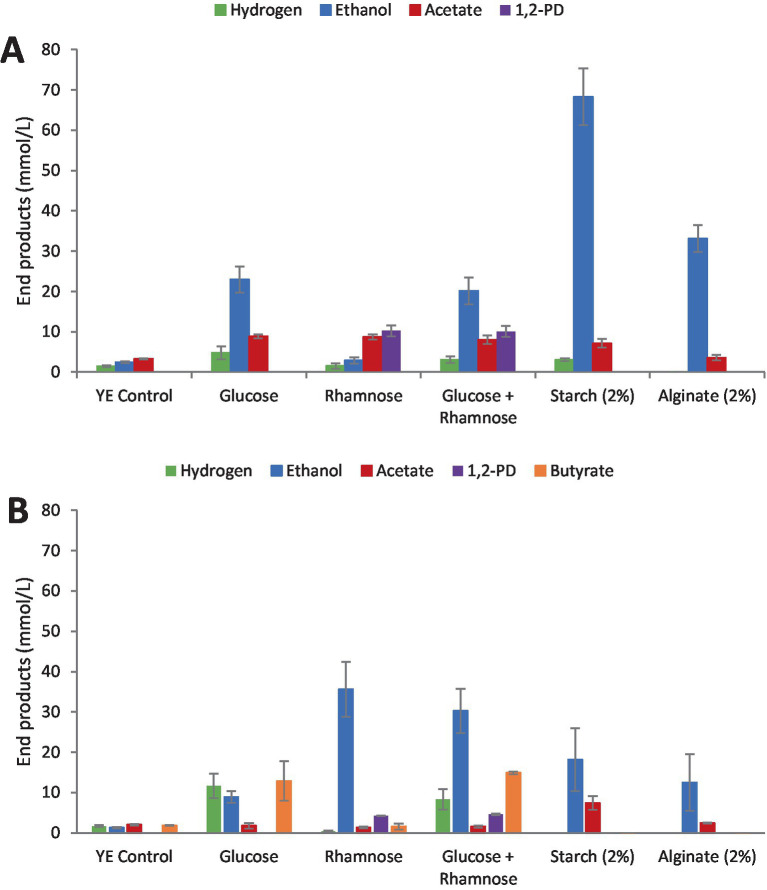
End product formation from model substrates and control (YE). **(A)** *Clostridium* strain AK1. **(B)** *C. beijerinckii*. Values represent the average of triplicates with standard deviation presented as error bars.

Fermentation end products of *Clostridium* strain AK1 when cultivated on glucose, rhamnose, a mixture of glucose and rhamnose, starch, and alginate are shown in Figure 3A. While alginate is not a component of *U. lactuca*, it is included here to fill an existing gap in the literature. The strain produced ethanol as the major end product from glucose metabolism with yields close to 1.1 mol ethanol/mol glucose as summarized in Figure 3A. During degradation of rhamnose, ethanol yields were generally lower, and formation of 1,2-PD was observed. When cultivated on a mixture of glucose and rhamnose, all end products were observed, but ethanol yields were lower as compared with growth on glucose alone.

*C. beijerinckii* produced mainly ethanol and butyrate from glucose, but more than three times higher ethanol concentrations were observed from rhamnose fermentation on average, in addition to 1,2-PD formation (Figure 3B). Low amounts of acetate and butyrate were observed during methylpentose degradation. During fermentation of a mixture of glucose and rhamnose, all fermentation products were observed with ethanol formation a little less than compared with fermentation of glucose only. Degradation of the polymeric substrates only resulted in the formation of ethanol and acetate.

Residual sugar(s) was not analyzed in the culture media after fermentation in this experiment. However, from the amount of end products analyzed it can be stated that *Clostridium* strain AK1 degraded about 55% of the rhamnose provided while *C. beijerinkii* degraded the sugar more or less completely.

### 3.4 Influence of culture conditions on L-rhamnose fermentations

**Figure 4:**
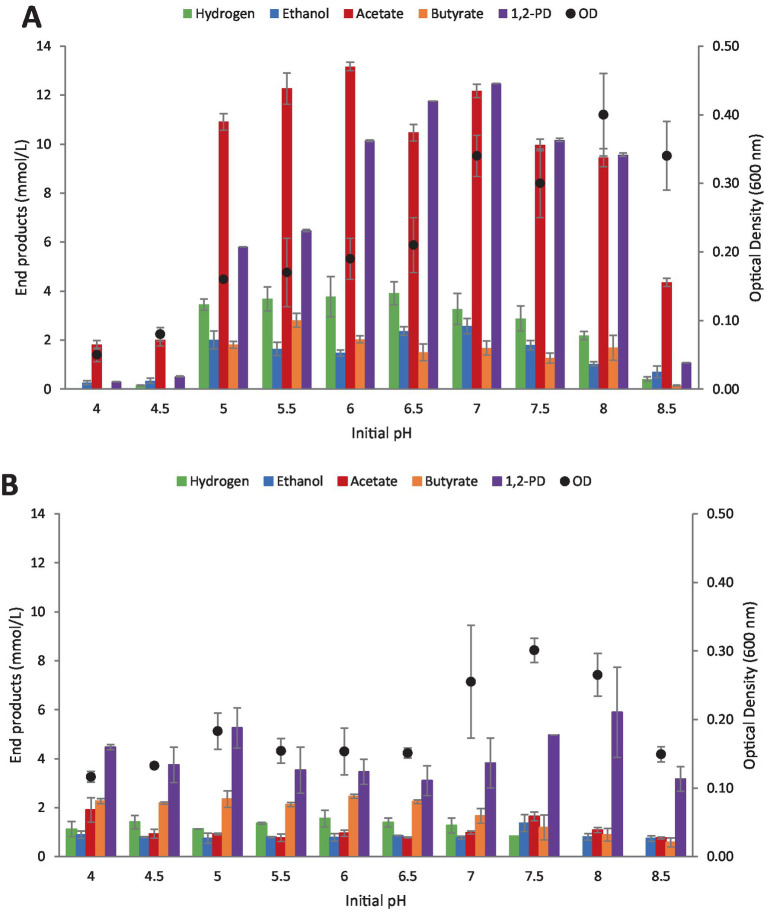
Influence of initial pH on rhamnose fermentation and control (YE). **(A)** *Clostridium* strain AK1. **(B)** *C. beijerinckii*. Values represent the average of triplicates with standard deviation presented as error bars.

In order to understand the influence of initial pH on end product formation from L-rhamnose both clostridia strains were cultivated on rhamnose (20 mM) over a period of 14 days at pH values ranging from 4.0 to 8.5. Both strains produced some 1,2-PD across each of the pH values examined (Figures 4A,B).

*Clostridium* strain AK1 was capable of rhamnose utilization between pH 5.0 and 8.0 with a maximum 1,2-PD titer of 12.5 mM (62.4% theoretical yield) obtained at pH 7.0 (Figure 4A) which is in accordance with a previously published study (Ingvadottir et al., 2018). Other end products observed at pH values between 5.0 and 8.0 were acetate (9.4–13.2 mM), ethanol (1.0–2.6 mM), butyrate (1.3–2.8 mM) and hydrogen (2.2–3.1 mmol/L).

*Clostridium beijerinckii* (DSM 791) showed end product formation at all pH conditions investigated. Highest 1,2-PD yields (5.9 mM) were observed at pH 8.0 but average differences in 1,2-PD yields across all pH values tested were low (Figure 4B). Other end products were acetate, ethanol, butyrate, and hydrogen. The amount of rhamnose utilized was highest at pH 8.0 with 30.9% corresponding to a 1,2-PD yield of 29.5%. The 1,2-PD yields based on 100% conversion of rhamnose were in general low (between 14.3 to 30.9%) but based on rhamnose degraded and 1,2-PD produced the ratio was around 1:1.

It is a well-known phenomenon that thermophiles in general are severely influenced by higher initial substrate concentrations. Thus, by increasing substrate concentrations, a clear trend is observed where the bacteria involved degrade less and less portion of the substrate. The main reason for this may be due to the partial pressure of hydrogen produced with higher substrate loadings or simply the lowering of pH in batch cultures. To investigate this phenomenon, *Clostridium* strain AK1 and *C. beijerinckii* were cultivated at different initial rhamnose concentrations (10–120 mM).

**Figure 5:**
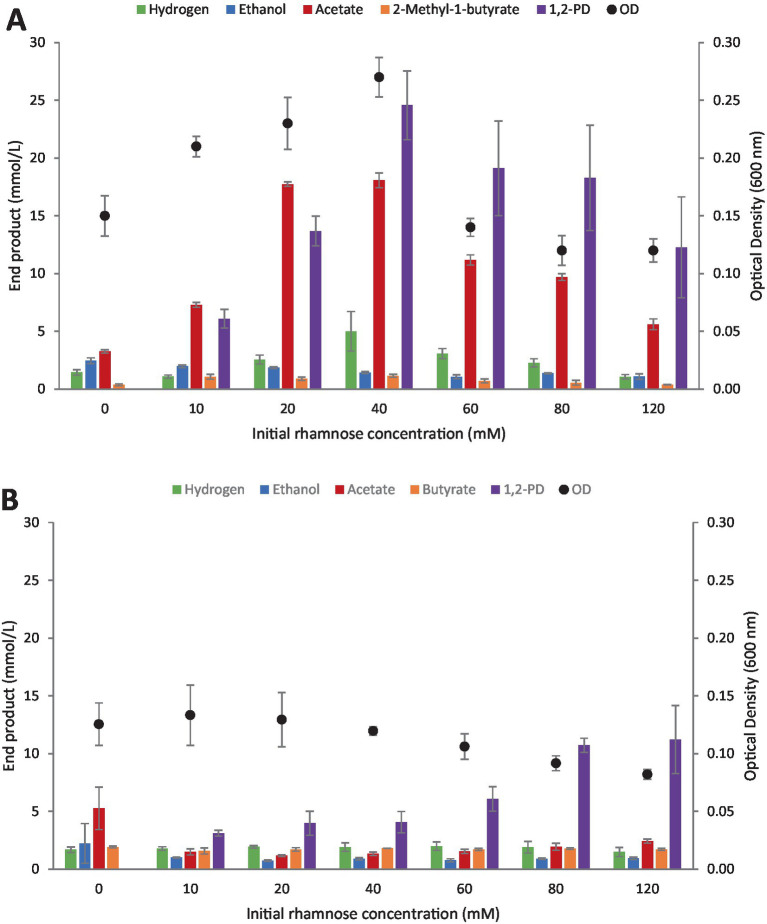
Influence of initial rhamnose concentration on its utilization. **(A)** *Clostridium* strain AK1. **(B)** *C. beijerinckii*. Values represent the average of triplicates with standard deviation presented as error bars.

The aforementioned trend was observed with both strains which were inhibited at low initial rhamnose concentrations, resulting in low substrate percent utilization as summarized in Figures 5A,B. At the lowest rhamnose concentration, 10 mM, between 45.9% (*C. beijerinckii*) and 78.3% (*Clostridium* strain AK1) of the methylpentose were utilized. These values decreased with increasing initial rhamnose concentrations and at 120 mM, only between 13.8 (*Clostridium* strain AK1) and 15.6% (*C. beijerinckii*) were degraded.

As before, the highest 1,2-PD titers were observed by *Clostridium* strain AK1 (highest at 40 mM rhamnose concentrations), reaching 24.6 mM. Other end products followed a similar trend as observed in the pH experiment described above.

### 3.5 Fermentation of *Ulva lactuca* hydrolysates

*Clostridium* strain AK1 and *Clostridium beijerinckii* were grown on BM diluted (5–70% v/v) *U. lactuca* hydrolysates (100 g/L) prepared under neutral (dH_2_O) and acidic (1% v/v H_2_SO_4_) conditions at 75 °C for 180 min. These hydrolysate preparation conditions were selected based on results from the evaluation of acid and base extraction of carbohydrates (see Sections 3.1 and 3.2). Undiluted hydrolysates prepared in water and under slightly acidic conditions resulted in rhamnose concentrations of 18.0 and 32.1 mM, respectively. Both strains were additionally grown on untreated *U. lactuca* meal (10% solid loading (w/v)). End products were quantified following 14 days of incubation at the T_opt_.

**Figure 6:**
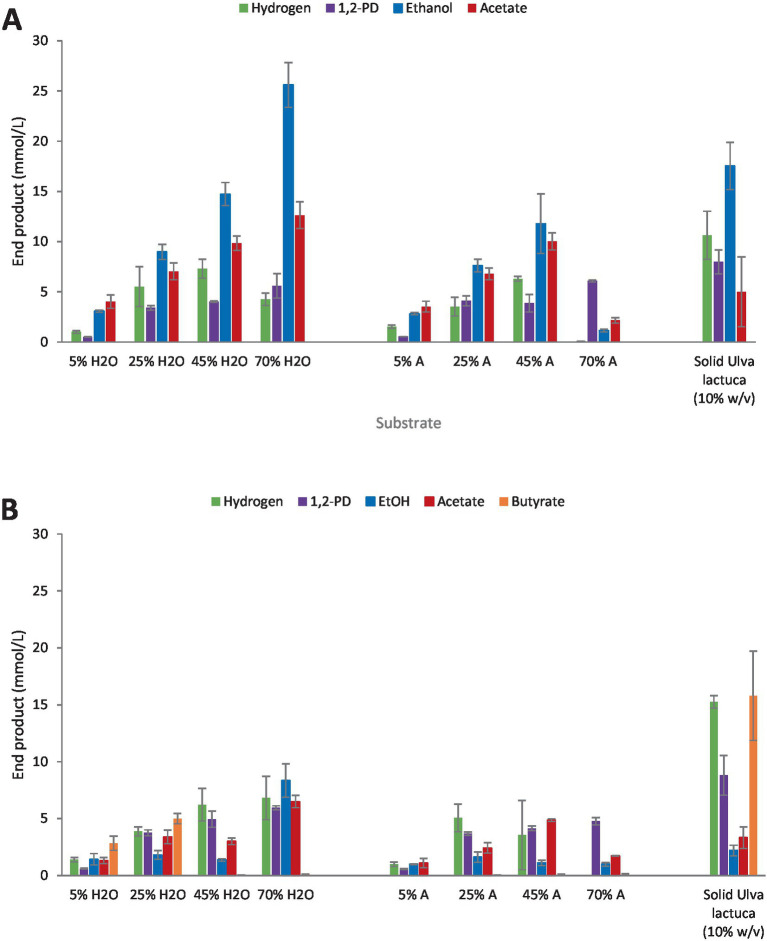
End product formation on *U. lactuca* hydrolysates [in water (H_2_O) and acid (A)] and untreated algae [solid *Ulva lactuca* (10% w/v)]. **(A)** *Clostridium* strain AK1, **(B)** *C. beijerinckii.* Values represent the average of triplicates with standard deviation presented as error bars.

As summarized in Figure 6A, *Clostridium* strain AK1 yielded the highest 1,2-PD titers when grown on untreated *U. lactuca* meal, or 8.0 mM which corresponds to 0.3 mol 1,2-PD/mol rhamnose assuming complete degradation. When grown on diluted hydrolysates prepared under neutral conditions, a positive correlation was observed between the increase in initial hydrolysate concentration and 1,2-PD, ethanol, and acetate concentrations (*r* = +0.96, *r* = +0.99, and *r* = +1.0 respectively), with strain AK1 reaching 53% of theoretical 1,2-PD yields when grown on 25% (v/v) hydrolysate. Ethanol and acetate concentrations ranged from 3.1–25.6 and 4.0–12.6 mM, respectively. In comparison, when grown on diluted hydrolysates obtained under acidic conditions, a similar correlation between end product and hydrolysate concentration was observed with the exception of 70% (v/v) where lower concentrations of end product formation were observed. Lower ethanol and acetate production were observed as compared with hydrolysates prepared under neutral conditions, ranging from 1.1–11.8 and 2.1–10.0 mM, respectively.

*C. beijerinckii* showed a similar behavior, producing highest amounts of 1,2-PD from undiluted (untreated) hydrolysate (8.8 mM) or 0.33 mol 1,2-PD/mol rhamnose, as shown in Figure 6B. Similarly, highest diol yields were obtained on 25% (v/v) hydrolysates, with 0.55 mol 1,2-PD/mol rhamnose for both hydrolysates prepared under neutral and acidic conditions. Other end products (ethanol, acetate, and hydrogen) were generally lower as compared with strain AK1 as seen in Figure 6B.

### 3.6 Kinetic study of carbohydrate fermentation by strain AK1

To investigate in more detail the fermentation kinetics of both sugars, *Clostridium* strain AK1 was cultivated on glucose (20 mM), rhamnose (20 mM), and a mixture of glucose and rhamnose (each a concentration of 20 mM).

**Figure 7:**
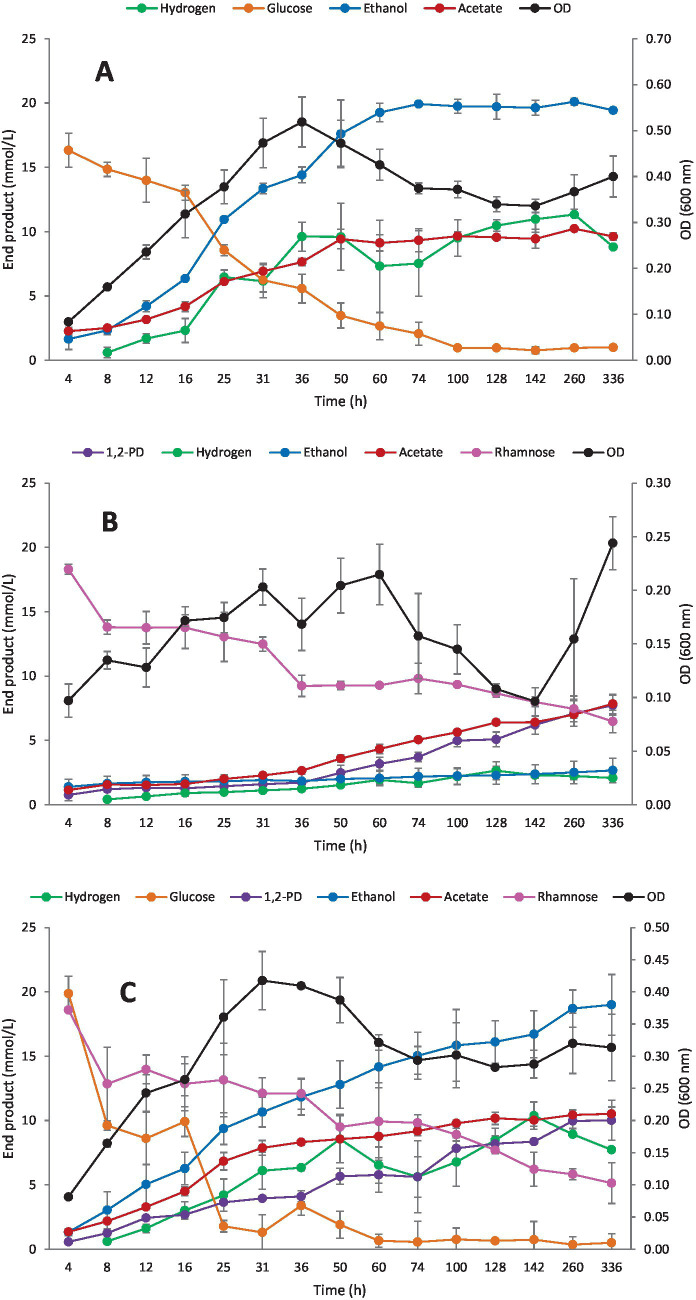
Kinetic end product formation of *Clostridium* strain AK1. **(A)** Glucose (20 mM), **(B)** Rhamnose (20 mM), **(C)** Glucose + rhamnose (each 20 mM). Values represent the average of triplicates with standard deviation presented as error bars.

The strain degraded more than half of the glucose during the first 24 h of incubation (Figure 7A). The maximum OD was 0.52 (36 h) and end product formation of ethanol, acetate, and hydrogen reached 19.4 mM, 9.6 mM, and 8.8 mmol/L, respectively. At the end of fermentation, almost all glucose had been degraded yielding the final stoichiometric equation as follows:


1.0Glucose→1.11EtOH+0.52Acetate+0.48Hydrogen

The relatively low carbon recovery may be explained by biomass formation and/or lactate production (the latter was not analyzed). During utilization of rhamnose as the sole carbon source, the growth was generally lower; about one third of the sugar was not degraded (Figure 7B). Additionally, during the first 48 h of fermentation, only a small fraction of the sugar was degraded, and small amounts of end products were observed. Final end products were ethanol (2.7 mM), acetate (7.8 mM), 1,2-PD (7.8 mM), and hydrogen (2.1 mmol/L). This yielded the following stoichiometric equation:


$$\begin{array}{c} 1.0\;\text{Rhamnose}\to 0.20\;\text{EtOH}+0.58\;\text{Acetate}+0.58\;1,2\\ -\mathrm{PD}+0.15\;\text{Hydrogen} \end{array}$$

Degradation of a mixture of glucose (20 mM) and rhamnose (20 mM) resulted in a similar growth rate compared with glucose alone (Figure 7C). However, glucose was degraded faster as compared with rhamnose, leading to a rapid increase in ethanol and acetate, whereas the formation of 1,2-PD lagged behind in correlation with low degradation rates of the methylpentose but gradually increased during the fermentation time. At the end of fermentation, 3.0 and 5.1 mM of glucose and rhamnose remained in the culture broth, respectively. At end of fermentation, final concentrations of ethanol, acetate, 1,2-PD, and hydrogen were 19.0 mM, 10.5 mM, 10.6 mM, and 7.7 mmol/L, respectively.

## 4 Discussion

The work described herein is the first report of the production of 1,2-PD by a moderately thermophilic bacterium from macroalgae hydrolysates, specifically *U. lactuca*. To date, Bikker et al. (2016), Potts et al. (2013), and Diallo et al. (2019) have reported 1,2-PD formation from *U. lactuca* using mesophilic *Clostridium* strains. Furthermore, this is the first report of possible alginolytic properties of *C. beijerinckii* strain DSM 791 and *Clostridium* strain AK1. To date, fermentation of alginate components has only been reported for *C. beijerinckii* strain DSM 6422 (Hou et al., 2017).

### 4.1 Evaluation of pretreatment and kinetics of hydrolysis

To identify the mildest conditions liberating the maximum concentration of L-rhamnose, preliminary sugar extraction from *U. lactuca* was performed under neutral, acidic, and basic conditions at temperatures ranging from 25 °C to 100 °C. To establish how long extractions should be conducted for in order to avoid a decrease in fermentable carbohydrate content while minimizing the production of inhibitory compounds, macroalgal extractions were repeated under selected conditions on a larger scale under chosen acidic conditions and the liberation of sugars followed over a period of 360 min. Additionally, a preparative scale extraction was performed at neutral and one chosen acidic condition.

The main components of *U. lactuca* biomass are sugars and proteins (Bikker et al., 2016). The total sugar content of the algae is about 24% of dry matter consisting of mainly glucose, rhamnose, and xylose. The amount of rhamnose analyzed in 10% (w/v) was maximally around 40 mM which corresponds to about 6.1 g/L. This is in correlation with what has been observed elsewhere (Bikker et al., 2016). Thus, *U. lactuca* has great potential as the raw material for 1,2-PD production via fermentation of a high yielding bacterial strain, producing up to 30 g/L.

At 0.1–2.5% v/v H_2_SO_4_, an increase in temperature was positively correlated with an increase in total sugars liberated after a 60 min extraction (*r = +* 0.8 for 0.1% and *r = +* 0.9 for 0.5–5% (v/v) H_2_SO_4_), although the resulting titers were up to 10-fold lower at 0–0.1% as compared with concentrations between 0.5 and 2.5% v/v H_2_SO_4_. This can be explained by the effective cleavage of glycolytic bonds from starch under acidic conditions. A similar trend was observed for the extraction of L-rhamnose, with a maximum concentration of 41 mM obtained after a 60 min extraction in 2.5% v/v H_2_SO_4_ at 75 °C. At the highest acid concentration, however, 5% H_2_SO_4_, a substantial decrease in total sugars was observed, most likely due to carbohydrate dehydration to compounds such as 5-hydroxymethylfurfuraldehyde by the acidic solution and elevated temperatures. However, a similar decrease in L-rhamnose yields was not observed. It is hypothesized that hexoses, such as glucose, are more quickly dehydrated into corresponding aldehyde species than deoxy sugars under increasing acidic conditions and temperature. This hypothesis was not experimentally tested although visual inspection of samples revealed a definite color change under increasingly acidic conditions and temperature; from clear to yellow to dark brown which could indicate Maillard reaction products from the reactions of the aforementioned sugar decomposition products and proteins present in *U. lactuca*. An increase in phenolics, which potentially have antimicrobial activity (Pérez et al., 2016), was observed with elevated acid concentrations and temperature. Total phenolics reached a maximum of approximately 370 *μ* mg/L after a 60 min extraction at 2.5% (v/v) H_2_SO_4_, 100 °C.

A clear shift in hydrolysis products was observed under basic conditions. Unsurprisingly, the recovery of total sugars was much lower than under acidic conditions as glycosidic linkages are more easily cleaved under acidic conditions. Similarly, the amount of protein liberated was much higher on average under basic conditions, as these conditions tend to favor the cleavage of the polypeptide bond (data not shown). The highest titers of L-rhamnose were systematically observed at 100 °C at all but two (dH_2_O and 0.1% (w/v) NaOH) extraction conditions. As under acidic conditions, the concentration of potentially antimicrobial phenolics increased with increased base concentration and temperature, reaching a maximum of approximately 656 mg/L after a 60 min extraction at 5% (w/v) NaOH, 100 °C. At all temperatures, for NaOH concentrations above 0.1% (w/v), the increase in total phenolics was strongly correlated with increased NaOH concentration (*r* = +0.97 in all cases). For these reasons, subsequent work focused on the use of acidic conditions.

To identify the optimum hydrolysis conditions for L-rhamnose liberation from *U. lactuca,* a series of kinetic experiments under acidic conditions were performed. Additionally, as it is well-known that non-enzymatic browning reactions in protein and sugar rich substrates increase with temperature and frequently include antibacterial products, it was decided neither to inspect the 100 °C extraction conditions, any of the basic conditions nor the most acidic conditions (5% (v/v) H_2_SO_4_) further. The reasoning for this was fourfold: firstly, the difference between L-rhamnose liberated after 60 min at 100 °C between 2.5% (v/v) H_2_SO_4_ and 5% (v/v) H_2_SO_4_ during the preliminary extraction was statistically insignificant (*p* < 0.05, data not shown). Secondly, the L-rhamnose liberated after 60 min at 75 °C and 2.5% (v/v) H_2_SO_4_ was significantly higher than at 5% (v/v) H_2_SO_4_ (*p* < 0.05). Thirdly, at temperatures below 100 °C, the maximum L-rhamnose recovered from basic extraction conditions was very low (5.7 mM solution). Fourthly, the aim of every extraction method should be that of a mild and energy efficient process, ruling out high temperatures (in this case 100 °C) and extractant concentrations (in this case 5% (v/v) H_2_SO_4_).

Likewise, the difference in L-rhamnose liberated under neutral conditions and 0.1% (v/v) H_2_SO_4_ was statistically insignificant (*p* > 0.05). Thus, the 0.1% (v/v) H_2_SO_4_ extraction condition was no further investigated. Thus, the kinetics of L-rhamnose, total reducing sugars, phenolic compounds and starch liberation were only examined for dH_2_O, 0.5, 1, and 2.5% (v/v) H_2_SO_4_. Finally, 75 °C was chosen as the acceptable extraction temperature as the increase observed in L-rhamnose extraction between 50 and 75 °C was statistically significant (*p* < 0.05, data not shown) for dH_2_O, 0.5, and 1% (v/v) H_2_SO_4._

An increase in the acidity of the solution was strongly positively correlated with an increase in total sugars liberated at 360 min (*r* = +1.0) and a clear increase in L-rhamnose concentration after 180 min at all acid concentrations. This positive correlation for L-rhamnose liberation, however, gradually decreased with increased acid concentration (*r* = +0.9 for dH_2_O but *r* = +0.7 for 2.5% (v/v) H_2_SO_4_). Contrary to brown and red algae, *U. lactuca* stores parts of its carbohydrates in the form of starch. Thus, in addition to L-rhamnose and total sugar analysis, starch was assayed. With increased acid concentration and time, a decrease in starch was observed, resulting in a total reducing sugar concentration increase. For example, at 2.5% (v/v) H_2_SO_4_, an increase in starch liberation was strongly correlated with time for the first 90 min (*r* = +0.9) but decreased to (*r* = −0.9) for the remaining extraction period (120–360 min). This suggests active starch hydrolysis at higher acid concentrations which correspond to the literature. Generally, the concentration of total phenolics increased with time, highlighting the need to limit extraction times to a minimum.

### 4.2 Fermentation of model substrates and effects of environmental factors on growth

The clostridia strains selected for the study herein have been previously noted to produce 1,2-PD from L-rhamnose (Ingvadottir et al., 2018; Ingvadottir et al., 2017; Diallo et al., 2019). The ability of these strains to utilize not only L-rhamnose but other components commonly found in biomass make them potential bioprocessing organisms for algal biomass although several knowledge gaps still exist some of which are partially addressed herein. *Clostridium* strain AK1 typically ferments glucose into a mixture of acetate, ethanol, and hydrogen (Orlygsson, 2012), while *Clostridium beijerinckii* further reduces acetate to butyrate. For referential purposes, these strains were also grown on starch and alginate as those are polymers frequently found in macroalgal biomass. As expected, when grown on rhamnose and a mixture of glucose and rhamnose, 1,2-PD yields of *Clostridium* strain AK1 remained the same on average while the formation of other end products increased in the presence of glucose.

To better understand the impact of specific culture parameters, in this case pH and initial substrate concentration, both strains were evaluated on rhamnose at various initial concentrations and pH. Changes in pH and initial substrate concentrations are known to cause shifts in end product formation during fermentation and/or to inhibit substrate utilization all together as has previously been depicted for *Clostridium* strain AK1 in the case of pH and D-glucose (Ingvadottir et al., 2018).

In general, strain AK1 is a more efficient 1,2-PD producer compared with *C. beijerinckii*, with yields often reaching more than 60% that of theoretical (compared with lower than 30% yields by *C. beijerinckii*). Interestingly, *C. beijerinckii* produced 1,2-PD at all pH values tested, but strain AK 1 showed highest yields on average between pH 6.0 and 8.0. Differences in pH did on average not influence the production of 1,2-PD by *C. beijerinckii* with yields reaching a maximum of 5.9 mM. A one-way ANOVA revealed a significant difference (*F*(9, 20) = [5.3], *p* < 0.001) within the groups with Tukey’s HSD finding means to be significantly different between pH 4.5–7 and 8, respectively, (*p* < 0.05), with the basic conditions favoring 1,2-PD production. Both strains seem to be inhibited by very low initial substrate concentrations, not being capable of fully degrading the lowest concentration of rhamnose (10 mM) used. On average, an increase in 1,2-propanediol production was observed for *C. beijerinckii* with increase in initial rhamnose concentration from 0–120 mM. However, a statistically significant difference was not observed between the highest initial concentrations of 80 and 120 mM. A one-way ANOVA revealed a significant difference (*F*(6, 14) = [12.9], *p* < 0.001) within the groups with Tukey’s HSD finding means to be significantly different between 10–60 mM and 80–120 mM, respectively (*p* < 0.05). This is common for fermentative bacteria, with the main explanation often being lowering of pH in the culture medium or accumulation of hydrogen in batch cultures.

### 4.3 Fermentation of *Ulva lactuca* hydrolysates and kinetic study on strain AK1

Degradation of glucose, rhamnose, and a mixture of glucose and rhamnose elucidated the degradation pattern for *Clostridium* strain AK1 on these substrates. The degradation rate of glucose as the sole carbon source was 0.33 mM/h with an ethanol production rate of 0.27 mM/h and a generation time of 8 h. Although rhamnose was degraded at similar rates as glucose (0.28 mM/h), its main end product, 1,2-PD, was produced at a much slower rate than ethanol during glucose fermentation. Surprisingly, when grown on a mixture of glucose and rhamnose, more 1,2-PD was generally produced as compared with rhamnose alone. It is noteworthy that degradation of rhamnose takes much longer than that of glucose and the formation of 1,2-PD from rhamnose increased steadily during the 336 h experimental period (14 days).

When grown on *U. lactuca* hydrolysates prepared under neutral (aqueous) and acidic conditions (1% v/v), an increase in hydrolysate concentration from 5% to 25, 45, and 70% (v/v) respectively led to a statistically significant increase in 1,2-PD formation (*p* < 0.05) by both strain AK1 and *C. beijerinckii*. In all instances, however, the 1,2-PD yields obtained from solid, non-pretreated *U. lactuca* were higher than that of hydrolysate batch cultures (*p* < 0.05).

Degradation of rhamnose by anaerobic bacteria has historically mostly focused on mesophilic *Clostridium* species. *C. beijerinckii* was shown to produce 0.44 mol 1,2-PD from one mole of rhamnose by Diallo et al. (2019). Similar yields were observed in *U. lactuca* hydrolysates with high initial rhamnose concentrations (238.7 mM) but less than 20% of the sugar was degraded. In the present study, *C. beijerinckii* generally showed higher yields of rhamnose conversion to 1,2-PD (up to theoretical yields; 1 mol 1,2-PD from 1 mol of rhamnose). The main reason for lower yields in the study of Diallo and coworkers as compared to the study herein is the fact that the strain produced not only 1,2-PD, but also propanol and propionate.

Bikker et al. (2016) reported yields of 0.3 to 0.4 mol 1,2-PD/mol rhamnose while Diallo et al. (2019) did not estimate the concentration of 1,2-propanediol at the end of hydrolysate fermentation despite reporting on the consumption of L-rhamnose. In the study described herein, yields were more than double that reported by Bikker et al. (2016), as *C. beijerinckii* produced a maximum of 0.8 and 0.5 mol 1,2-PD/mol rhamnose from diluted water and acid prepared hydrolysates, respectively. Strain AK1 showed similar yields as *C. beijerinckii* in present study, producing 1 mol of 1,2-PD from one mole of rhamnose as has also been published previously (Ingvadottir et al., 2018). Similar yields were also observed when the strain was grown on *U. lactuca* hydrolysates.

Based upon these results, it is likely that additional means for hydrolyzing ulvan to rhamnose requires either more aggressive hydrolysis, perhaps using a combination of alkaline conditions for the removal of the sulfate esters and mild acid hydrolysis to cleave the glycosidic bond, or enzymatic tools for the selective hydrolysis of ulvan in the presence of other polysaccharides. The latter option, given the slow fermentation kinetics of systems containing both glucose and rhamnose, may result in higher 1,2-PD titers. Future work should focus on the development of these approaches, in the context of a cascading biorefinery concept centered around *Ulva* biomass.

## 5 Conclusion

This work explores the hydrolysis and fermentation of *U. lactuca* to potentially useful end products such as ethanol and 1,2-propanediol by the moderately thermophilic *Clostridium* strain AK1. The hot acid hydrolysis (2.5% v/v H_2_SO_4_ at 100 °C) of *U. lactuca* yielded the highest monosaccharide content. Strain AK1 was able to ferment a hot water extract of *U. lactuca* to 17.3 mM (0.18 g/g) ethanol and 1.7 mM 1,2-propanediol (0.03 g/g) after 60 h of fermentation. The production of modest 1,2-propanediol titers on single substrates (rhamnose) compared to the relatively low yields on macroalgal hydrolysates suggest that some form of inhibition of the desired pathway may be at work. These findings demonstrate the utility of simple pretreatment strategies for the fermentation of *U. lactuca* highlighting the need for specific enzymatic tools to achieve a degradation of methylpentose-containing polysaccharides.

## Data Availability

The raw data supporting the conclusions of this article will be made available by the authors, without undue reservation.
